# Modeling Group Behavior to Study Innovation Diffusion Based on Cognition and Network: An Analysis for Garbage Classification System in Shanghai, China

**DOI:** 10.3390/ijerph16183349

**Published:** 2019-09-11

**Authors:** Junjun Zheng, Mingyuan Xu, Ming Cai, Zhichao Wang, Mingmiao Yang

**Affiliations:** Economics and Management School, Wuhan University, Wuhan 430072, China

**Keywords:** bounded rational individual, group structure, group behavior, scale-free network, diffusion of innovation, garbage classification

## Abstract

In real life, garbage has caused great pollution to the environment. A garbage classification system is an effective way to manage this issue, and is an innovation in Shanghai, China. Innovation diffusion is the topic of this paper. This study uses a mathematical statistics method to formulate individual bounded rationality, and uses the specific graph structure of a scale-free network to characterize group structure. Then, a model of group behavior is constructed and the simulation experiment is run on the Python platform. The results show that: (1) In the case of general cognitive ability and high value innovation, most individuals in the group will accept the innovation in the process of innovation dissemination in a garbage classification system after several rounds of the game; (2) it is more helpful to improve the cognitive ability of individuals and the true value of innovation for the diffusion of innovation; and (3) the larger a group, the greater the scope of innovation diffusion and the more time is needed. It is helpful to expand the scope and reduce the time of innovation diffusion by increasing connections among individuals. The innovation of this study is the characterization of individual bounded rationality, which has a certain theoretical value. Meanwhile, the research results of this paper have important practical significance for the promotion of garbage classification, which can be used to popularize the concept of garbage classification.

## 1. Introduction

The impact of pollution from garbage on the environment has become increasingly serious, and is causing major environmental problems. Therefore, it is necessary to take effective measures to manage garbage. Since 1 July 2019, a compulsory garbage classification system has been implemented in Shanghai, China, through legislation. Compared with previous garbage classification management, this system is an innovation that will have a profound impact on peoples’ lives. However, different people have bounded cognition of the innovation, and it is difficult to change living habits, so that the degrees of acceptance for the innovation are different. Therefore, how the innovation can effectively spread in the group, and the influence of individual bounded rationality and group structure on diffusion are the focus of this paper.

Firstly, the analysis of diffusion of innovation in groups is based on the assumptions of individual bounded rationality. This more closely resembles reality because, in real life, the behavior of each individual is influenced by their own cognition. The specific manifestation is that the perception of the same individual for the same thing in a different time and space may make a significant difference. Therefore, the analysis and expression of individual bounded rationality is the focus of the modeling and also the innovation of this paper. Mathematical statistics will be considered to describe the individual bounded rationality.

Secondly, each individual does not exist in isolation but lives in a society with complex social relationships, where the interaction among individuals is essential. Thus, individuals will also be affected by the social environment. When individuals interact with others, individuals connect and influence each other through social relationships. Social relationships constitute a complex social network over time. At same time, individuals form a group due to the connections of complex networks, and the group structure has an important impact on the spread of innovation in group. Thus, group structure should be depicted by a certain law. The scale-free network, which is closer to reality, is a common solution for social network analysis. Therefore, this paper will use a scale-free network to depict the group structure. At the same time, individuals are affected by their neighbors in the group, which is very important to the spread of innovation. Therefore, this paper will use a coordination game, a common model to analyze group behavior, to depict this influence.

Finally, multi-agent simulation technology is used to simulate the group behavior based on individual bounded rationality and group structure. The experiment is carried out on the Python platform, which is a common tool to simulate the propagation of innovation. 

The rest of this paper is organized as follows. Section 2 contains a literature review. In Section 3, the model of group behavior is constructed and the analysis indicators are given. In Section 4, the simulation experiment is designed. In Section 5 the above experiment is simulated, and the results are analyzed. The last section summarizes the full text and looks forward to future work.

## 2. Literature Review

This section mainly refers to the related research in the following two areas: group behavior and multi agent simulation.

### 2.1. Group Behavior

In the process of interacting with others in a complex network, interactions usually reinforce the initial views of the individuals in the group, which is called group polarization. For example, in the community, individuals with similar ideas will usually gradually converge, so that their shared views are strengthened. This also coincides with the view of the ancient Chinese saying “Similar people always come together”. If individuals with hostile personalities or dissatisfaction gather in groups to communicate with each other, then a criminal gang may emerge. With the development of the Internet, the restriction of the region and the separation of the society are broken, and individuals with the same character, purpose, interest, view, etc., are more easily united. At the same time, the resulting polarization is also increasing. Group polarization is one example of a group behavior phenomenon, such as conformity and herd behavior, which are also produced by the interaction of individuals in a group. Not only does the group have an impact on the individual, but the individual also acts on the group in turn, affecting the group in which it belongs, which is commonly known as minority influence.

Scholars at home and abroad have made some explanations for the causes of conformity, herd behavior or minority influence. In order to explore the impact of group discussion on jury decisions, moral judgments, individual perceptions, judgments, attitudes, negotiations and adventure, experimental research has found that group polarization usually occurs. Reasons for this phenomenon are explained by constructing a conceptual solution based on comparisons among individuals and information in [1]. Reference [2] studies the transformation of individual choice and the phenomenon of group polarization from the perspective of social psychology. When individuals interact with each other, differences between the final opinions and the initial opinions will lead to the transformation of individual choice. Unbalanced interpersonal relationships in the group are seen as the main reason for the transformation of individual choice. The interpersonal relationship network and the individual position in the network can explain the individual choice transformation and group polarization to some extent. Reference [3] focuses on the influence of confirmation deviation on group polarization. It changes the network structure by constructing a mathematical model and setting rules to study the network debate and related polarization dynamics. Through numerical simulation, it is found that two stable final viewpoints will coexist. In [4], through the construction of the dynamic trust model, the effect of trust cognition on the cooperation strategy choice is studied separately in the situations of one-time, limited and infinite number of prisoner dilemmas. From the perspective of group characteristics, [5] studies the phenomenon of network group polarization in social media, and analyzes the “group characteristics” and “group extreme opinions” by using the CORREL function, with results showing a correlation between them.

The above literature review highlights studies on the mechanism of group behavior. However, there are few studies on the analysis of group behavior from the individual level, and few studies consider the influence of individual bounded rationality and group structure on group behavior at the same time.

### 2.2. Multi Agent Simulation

Because of the complexity and uncertainty of social networks, it is difficult to carry out innovative communication experiments in practice. Fortunately, multi-agent simulation is a powerful tool that can simulate the real situation and has been used in different fields.

Reference [6] outlined an interpretation of the phenomenon of group polarization. Based on the cognitive hypothesis of related belief consistency, the fusion of different groups and group polarization are generated through simulation. In [7], investor conformity behavior and the influence of network structure on the stock market are discussed, and a simulation experiment of the stock market is carried out by cellular automaton. Reference [8] builds a complex system of a securities market from three aspects: trader, stock and information. Based on the complex network theory, the formation mechanism of herd behavior is studied by means of simulation experiment. Reference [9] studies the optimal response strategy of the government based on a scale-free network model. The simulation results show that the degree of individual conformity has a great influence on the speed of dissemination of terrorism information in a network and the optimal strategy of the government.

Therefore, in this paper, the group is considered to be composed of several unorganized bounded rational individuals. It uses a mathematical statistics method to formulate individual bounded rationality, and the specific graph structure of the complex network to analyze how individuals are affected by their neighbors. Thus, the spread of innovation from the perspective of group behavior can be studied.

## 3. Modeling

This section mainly consists of three parts: Firstly, by combining the participants and processes of communication, this paper establishes the conceptual model. Secondly, based on the conceptual model, parameters are defined and the model of group behavior is constructed. Finally, the analysis indicators are selected to study the dynamic diffusion of innovation in garbage classification.

### 3.1. The Conceptual Model

**Figure 1:**
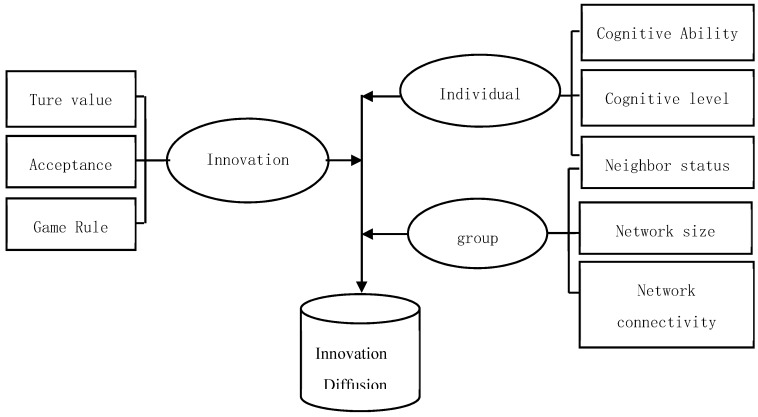
Conceptual model.

Because of limited cognitive ability, the individual has limited rationality. When the innovation of a garbage classification system is implemented, this innovation has a true value, and some individuals accept it, while others do not. At the same time, individuals will hold a certain degree of cognition for this innovation, which is called the cognitive level. In the process of individual interaction, a complex social network is gradually formed, which has a certain scale and connection degree. Individuals are not only affected by the group in the whole network, but also affect the group. Individuals change their behavior according to the behavior status of their neighbors and certain rules. The conceptual model is shown in Figure 1.

### 3.2. The Model of Group Behavior

According to the above description, the model of group behavior is designed. The whole model consists of three parts: the individual model, group structure and game rule.

#### 3.2.1. The Individual Model

**Figure 2:**
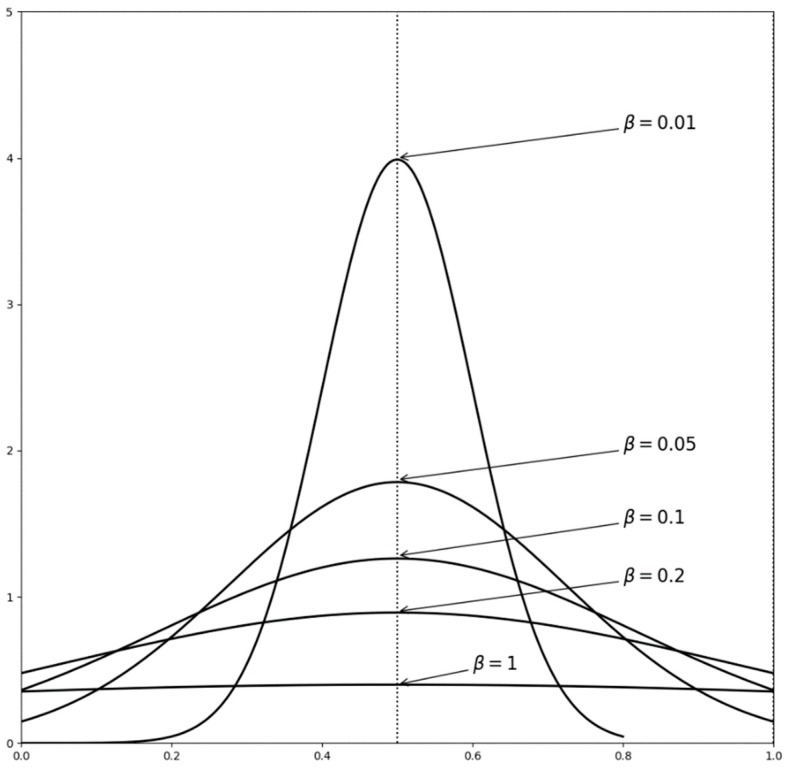
The diagram of the relationship between *β* and $\mu_{0}$.

Because individual cognitive ability is limited, it may lead to cognitive deviations. The characterization of bounded rational individuals begins with cognitive deviation. Thus, the parameters of the bounded rational individual are defined as follows:

1. σ: represents cognitive ability of the individual, where σ∈(0,1), which means that the cognitive ability of the individual is gradually enhanced from 0 to 1.
2. $\mu_{0}$: represents true value of innovation.
3. $\mu_{i,t}$: represents cognitive level of individual *i* at time *t*, where $\mu_{i,t}\in (0,1)$, which reflects the degree of individual cognition. In this measure, 0 indicates that the individual has no cognition about the innovation and 1 means complete cognition.
4. β: represents cognitive deviation. In order to facilitate the analysis, according to the objective situation, the related functional relationship between cognitive deviation β and cognitive ability σ is established as:
  (1) $$\beta =\frac{1}{\sigma }-1$$
5. g(x): represents a probability density function. At time *t* = 0, the cognitive level of individual *i* about the innovation is $\mu_{i,0}$, which obeys a distribution with a probability density function g(x) expressed as:
  (2) $$g\left(x\right)=\left\{ \begin{array}{ll} \frac{f_{1}(x)}{F_{1}(1)-F_{1}(0)}, & 0<x\leq 1\\ 0, & \mathrm{others} \end{array}\right.$$
  In the formula above, $f_{1}\left(x\right)$ obeys a normal distribution $N(\mu_{0},\beta^{2})$, whose probability density function is shown as:
  (3) $$f_{1}(x)=\frac{1}{\sqrt{2\pi }\beta }\exp(-\frac{{\left(x-\mu_{0}\right)}^{2}}{2\beta^{2}})$$
  The stronger the individual cognitive ability, the smaller the cognitive deviation. That is, the smaller the variance β, the closer the cognitive level of the individual is to the true value $\mu_{0}$, which is consistent with the actual situation in reality, as shown in Figure 2.
6. $w_{i,t}$: represents state set of individual *i*, where $w_{i,t}=\left\{0,1\right\}$, in which 0 means that the individual does not accept the innovation and 1 means the innovation is accepted.

#### 3.2.2. The Group Structure

A group is organically composed of many individuals and their interconnections. Individuals observe their neighbor state, which can be called the signal observed by the individual. However, the signal of each individual may be different, assuming that the signal is not transmitted among individuals, so it is difficult for individuals to observe or understand the signal obtained by other individuals. Thus, the construction of group structure is expressed as follows:

1. B: represents a set of *n* individuals in the group, where B={1,2,3…,n}. $B_{i}$ is a neighbor set of individuals.
2. G: represents a network of individuals, where G=(A,V), in which ***A*** is a set of directed edges and $V=\left\{v_{1},v_{2}\ldots ,v_{n}\right\}$ is a set of nodes in the network. If $v_{i}$ can observe $v_{j}$, there should be a pointing edge a(i,j)∈A from $v_{i}$ to $v_{j}$.
3. $S_{i,t}$: represents a status set of all nodes at time t, where $S_{i,t}=\left\{w_{1,t},w_{2,t},\ldots ,w_{n,t}\right\}$.
4. $q_{i,t}$: represents a proportion of neighbors who accept innovation, which means the whole neighbor status of individual *i* at time t, where the expression can be shown as:
  (4) $$q_{i,t}=\frac{\sum_{j\in Bi}w_{j,t}}{\left|B_{i}\right|}$$

#### 3.2.3. The Game Rule

In a social network, if node i and j interact, there will be an edge connected, and the action of one individual may be affected by the behavior of another Thus, each node has two optional strategies, A and B. According to the coordination game [10], the game rule is shown as: 

If i and j choose strategy A at the same time, then they get returns a>0, respectively; 

If i and j choose strategy B at the same time, then they get returns b>0, respectively; 

If i and j choose different strategies, then they both get returns 0. 

If a node in the network has a total of d neighbors, the proportion of who selects strategy A in the neighborhood is q, and the proportion of who selects strategy B is 1−q. Thus, if the node selects strategy A, the node will get returns aqd. If the node selects strategy B, the node will get returns b(1−q)d. When aqd> b(1−q)d, that is $q>\frac{b}{a+b}$ or $\frac{a}{a+b}>1-q$, the individual should select strategy A. 

The cognitive level $\mu_{i,t}$ of individual i at time t is the same as $\frac{a}{a+b}$, and $q_{i,t}$ is the proportion of neighbors who accept innovation. Thus, the rule is shown as:

(5) $$\mu_{i,t}\geq 1-q_{i,t}$$

Then, the individual changes their original behavior, namely $w_{i,t}=1$.

### 3.3. The Indicators for Analysis

In order to study innovation diffusion and the influence of cognitive ability and group structure on diffusion, two indicators are considered for analysis, as shown below.

1. $R_{t}$: represents the extent of innovation diffusion in the network, and is expressed by the proportion of individuals that accept innovation to the total number of individuals in the network. The formula is shown as:
  (6) $$R_{t}=\frac{\sum_{i=1}^{n}w_{i,t}}{n}$$
  When $t=t_{total}$, the innovation acceptance proportion is the final acceptance proportion.
2. T: represents the time required for the proportion of innovation acceptance to reach stability, and characterizes the propagation speed of innovation in a network. The formula is expressed as:
  (7) $$T=\inf\left\{t|R_{t}=R_{t+1}=\cdots =R_{t_{total}}\right\}$$

## 4. The Simulation Experiment Design

### 4.1. Initial Setting

Based on the above model, five simulation test groups are set respectively in the diffusion process of innovation and each group is divided into five cases.

In order to be closer to reality, this paper use the BA scale-free network to depict the group structure, which was proposed by Barabasi and Albert in 1999 [11]. The degree of nodes obeys a power-law distribution. The number of nodes in group is *n* = 100 and the degree of node is *l* = 2. The total time is 100. Initial settings are shown in Table 1.

### 4.2. Simulation Process Design

**Figure 3:**
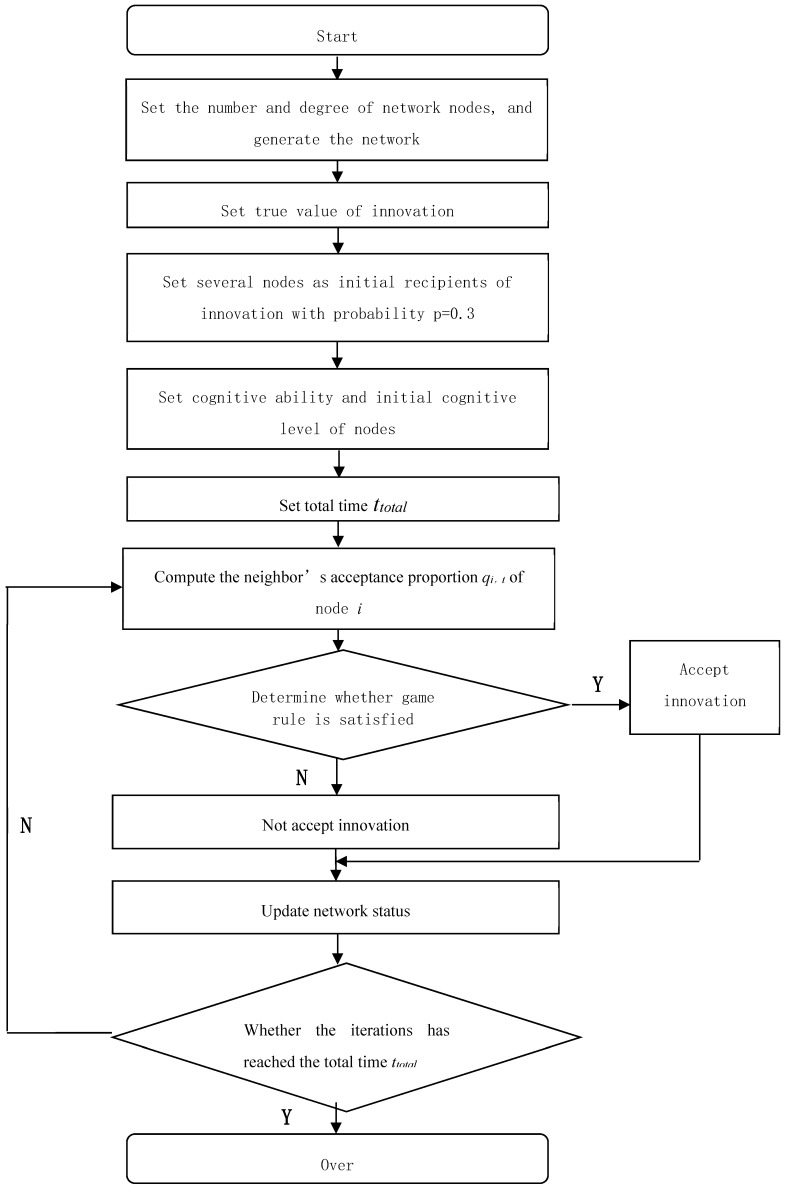
Simulation process diagram.

The simulation process is shown in Figure 3.

### 4.3. Description and Treatment of Random Error in Simulation Model

For the simulation model in this paper, there are some errors in the simulation results, which are mainly caused by the randomness of some parameters generated in the simulation process. The randomness is mainly reflected as follows:

1. When several nodes are set as recipients of innovation at the initial time, each node is set as the recipient according to the probability *p* = 0.3.
2. When the cognitive ability is set, each node’s cognitive level of innovation obeys the distribution with probability density g(x).

In order to reduce the influence of the error caused by randomness, the simulation experiment is repeated 50 times under each parameter condition, and we take the average value as the final simulation result.

## 5. Results and Analysis of Innovation Diffusion

In real life, the cognitive ability of individuals is not equal. Innovation diffusion in a complex network is simulated based on different cognitive abilities.

### 5.1. The Diffusion of Innovation

**Figure 4:**
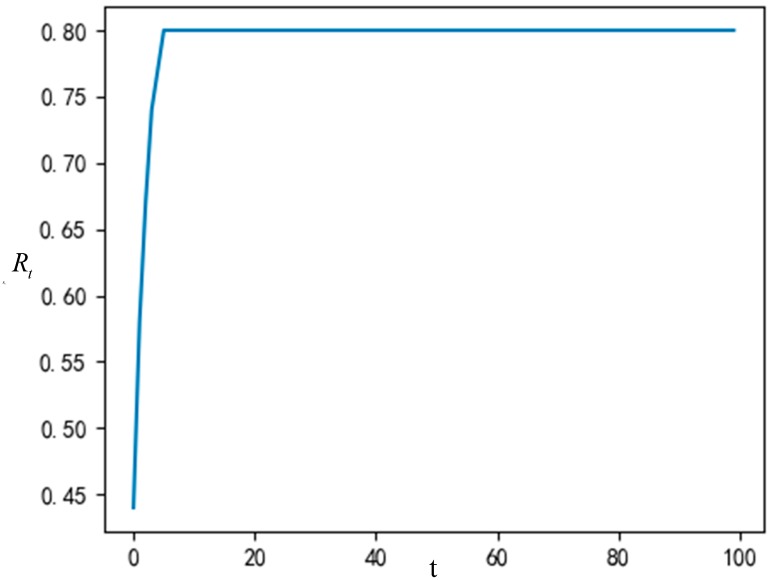
The proportion of nodes accepting innovation.

**Figure 5:**
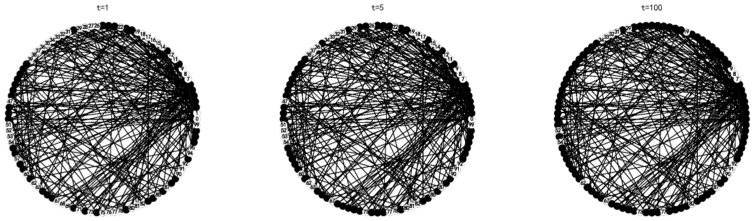
The status of the group at t = 1, t = 5, t = 100.

Shanghai is a big city with a high degree of civilization. In order to more accurately reflect reality, this paper assumes that the individual has a certain cognitive ability, namely σ=0.5, and the innovation of the garbage classification system is a high value innovation, that is, $\mu_{0}=0.9$. Then, the proportion of nodes accepting innovation, the change of node strategy and the node state in the network at different times are demonstrated in Figure 4 and Figure 5.

It can be observed from the Figure 4 above that when the time is t = 5, the network reaches a stable state, and the proportion of nodes accepting innovation is stable at 80%. It shows that 80% of the individuals in the group will accept the innovation in the process of innovation dissemination of the garbage classification system after several games.

### 5.2. Analysis of Cognitive Ability on Innovation Diffusion

The influence of cognitive ability σ and true value $\mu_{0}$ on the proportion *R_t_* of innovation and network stability time T is analyzed below.

#### 5.2.1. Influence of σ and $\mu_{0}$ on Diffusion Extent

The data obtained from running the simulation are shown in Table 2, which indicates the final acceptance proportion *R_t_* of the innovation in the network at different cognitive abilities σ and different true values $\mu_{0}$.

**Figure 6:**
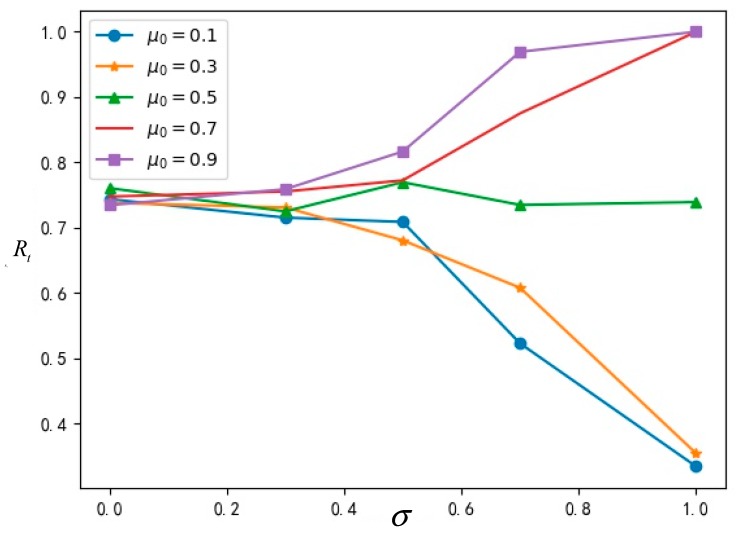
The relationship diagram among σ,$\mu_{0}$ and *R_t_*.

In order to analyze the relationship among cognitive ability σ, true value $\mu_{0}$ and the proportion *R_t_* of innovation acceptance more intuitively, a broken line diagram is shown in Figure 6 according to the data of the simulation results in the Table 2 above.

As can be seen from the above Figure 6, when the cognitive ability is at a lower level, the acceptance proportion of the innovation is less affected by the true value $\mu_{0}$; as the cognitive ability gradually increases, the acceptance proportion of the innovation is greatly affected by the true value $\mu_{0}$. This shows that when the cognitive ability of a group is insufficient, the true value of innovation has little influence on innovation diffusion and, when the cognitive ability of a group reaches a certain level, the true value of innovation will greatly affect innovation diffusion. Thus, an innovation with a high value has a greater effect on diffusion, and an innovation with a low value can be better identified in the group, whose influence on diffusion is also limited to a lower degree.

#### 5.2.2. Influence of σ and $\mu_{0}$ on Diffusion Speed

The data obtained from running the simulation are shown in Table 3, which indicates the final time to stability T of innovation diffusion in the network at different cognitive abilities σ and different true values $\mu_{0}$.

**Figure 7:**
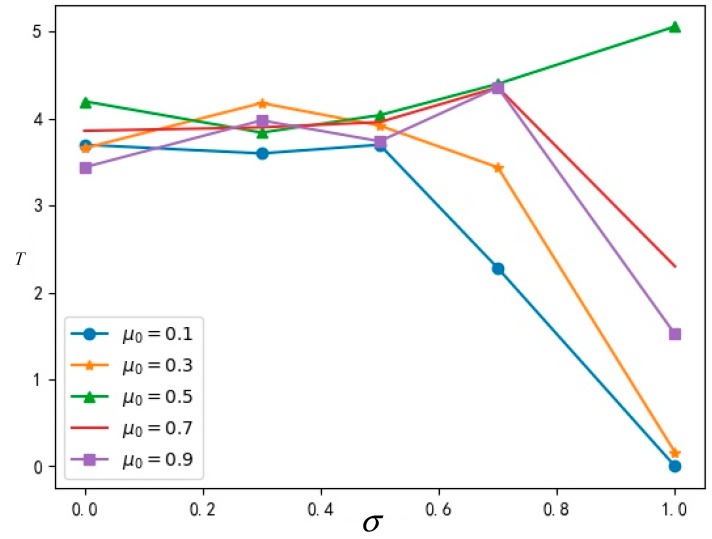
The relationship diagram among σ,$\mu_{0}$ and T.

In order to analyze the relationship among cognitive ability σ and the true value $\mu_{0}$ and the final time to stability T of innovation diffusion more intuitively, a broken line diagram is drawn in Figure 7 according to the data of the simulation results in the Table 3 above.

It can be seen from the above Figure 7 that, except for the case of $\mu_{0}=0.5$, the time of network stability decreases with the increase of cognitive ability. When the cognitive ability of the group generally rises, the propagation of innovation acceptance in the group can become stable more quickly. Namely, when the cognitive ability of the group is at a higher level, the value of innovation is recognized and the network become stable more easily.

When $\mu_{0}=0.5$, it means that the value of innovation is very close to the value of the original event, so it will take a longer time to distinguish the innovation from the original event in the group network. Namely, when $\mu_{0}=0.5$, it will take longer to reach a stable state.

### 5.3. Analysis of Group Structure on Innovation Diffusion

In order to explore the influence of different network structures on innovation diffusion in the network, the number and the degree of network nodes are set as dependent variables in order to generate networks with different structures. Then, we run the simulation program and record the acceptance proportion of innovation and the time required for network stability in each case.

#### 5.3.1. Influence of Node Degree on *R_t_* and T

**Figure 8:**
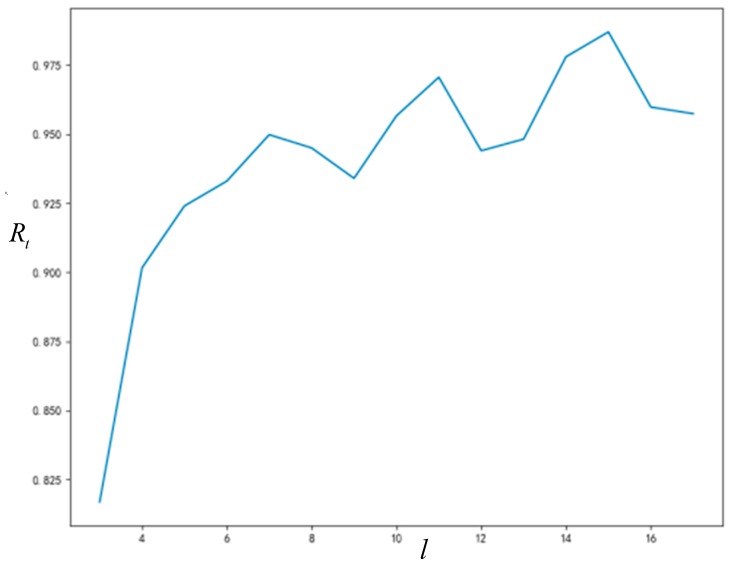
The relationship between node degree and *R_t_*.

**Figure 9:**
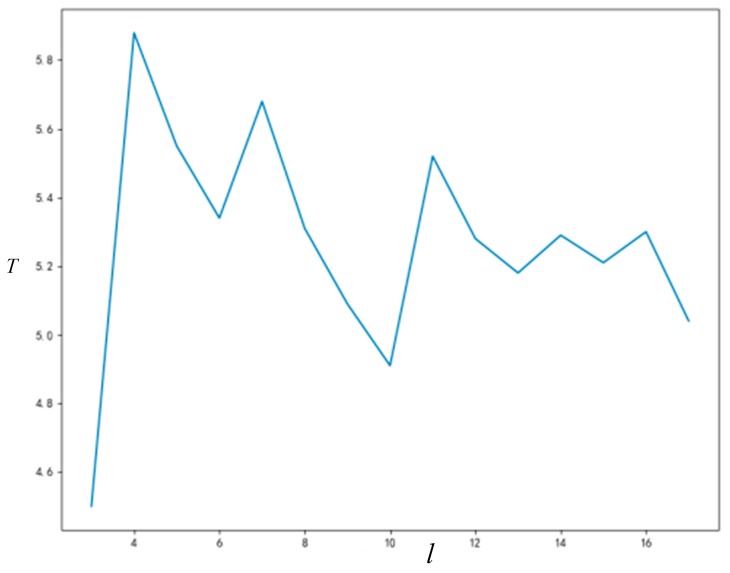
The relationship between node degree and T.

As can be seen from Figure 8 and Figure 9, the final acceptance proportion of innovation will increase with the increase of network connection degree, and the time required for network stability will decrease with the increase of network connection degree. Due to the randomness of some parameters generated in the simulation process, the final acceptance proportion of innovation and the time required for network stability will not change monotonously with the increase of network connection degree; however, this does not affect the trends that we can observe. 

#### 5.3.2. Influence of Node Number on *R_t_* and T

**Figure 10:**
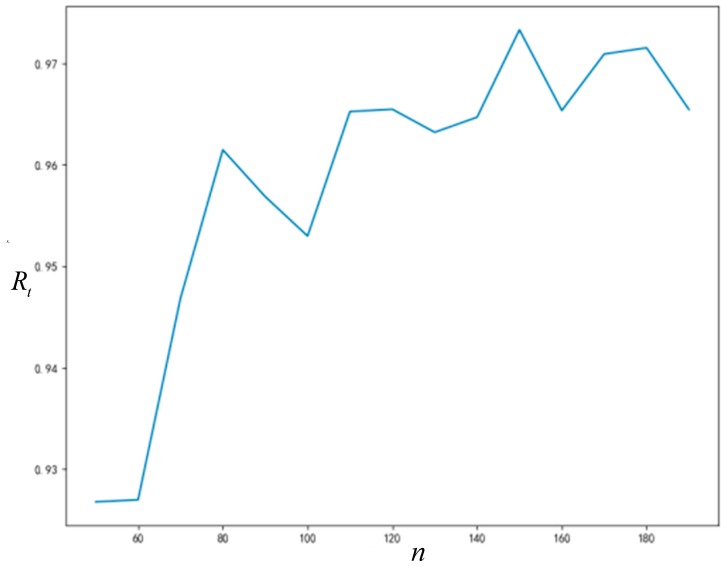
The relationship between node number and *R_t_*.

**Figure 11:**
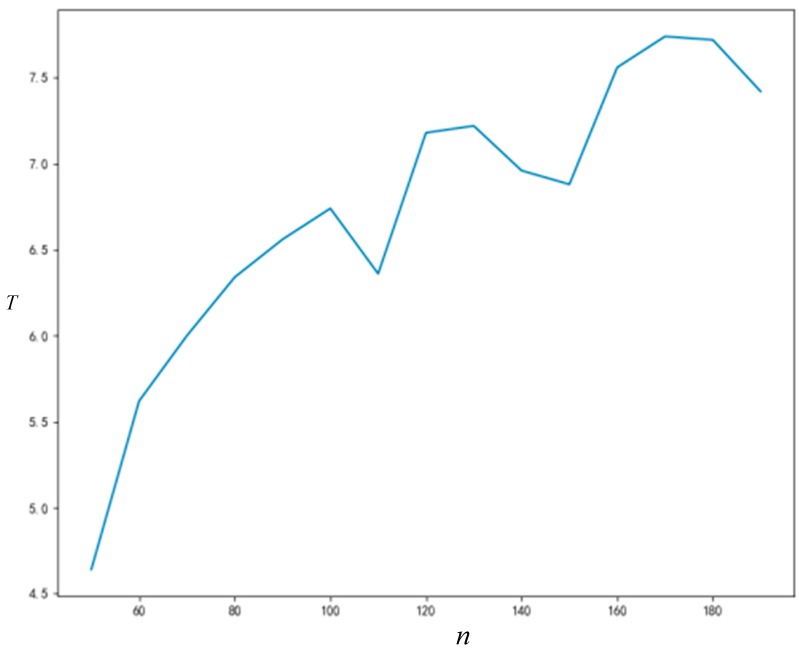
The relationship between node number and T.

As can be seen from Figure 10 and Figure 11, the final acceptance proportion of innovation and the time required for network stability will increase with the increase of network size (number of nodes). The final acceptance proportion of innovation and the time required for network stability will also not increase monotonously with the increase of network size, and the non-monotonic changes are also caused by the randomness of some parameters generated in the simulation process. Nonetheless, they continue to show a significant upward trend. If it is necessary to reduce the influence of the error caused by the randomness, repeating the simulation experiment more times can improve the situation.

## 6. Conclusions

This paper discusses the problem of innovation diffusion of a garbage classification system based on the constructed group behavior model in the context of individuals with bounded rationality and scale-free networks. From the simulation results, it is important for governments to promote the diffusion of innovation by taking targeted measures. Furthermore, the following conclusions can be obtained:

1. In the case of general cognitive ability and high value innovations, most individuals in the group will accept the innovation in the process of innovation dissemination of a garbage classification system after several rounds of the game.
2. It is more helpful to improve the cognitive ability of individuals and the true value of innovation for the diffusion of innovation.
3. The larger a group, the greater the scope of innovation diffusion and more time is required. It is helpful to expand the scope and reduce the time of innovation diffusion by increasing connections among individuals.

This paper focuses on characterizing the bounded rationality of individuals, using the scale-free network to characterize the group structure, and studies innovation diffusion among individuals in the network. However, there are still some shortcomings in this research. Therefore, in future research, we will conduct more in-depth investigations considering the following aspects:

1. It is meaningful to explore more factors influencing individual bounded rationality, which can be depicted by establishing relevant models using behavioral experiments, econometrics and other theoretical methods, and to study innovation diffusion based on these bounded rational individuals.
2. Whether the interaction among individuals and the updating of cognitive levels will have an impact on innovation diffusion is also a topic worthy of discussion.

## Figures and Tables

**Table 1:** Initial setting.

The Category of Parameters	Parameters	Initial Setting
Parameters of nodes	σ	*σ_i_* = 0.00001, 0.3, 0.5, 0.7, 0.99999
	*μ_i,_*_0_	*μ_i,_*_0_*~g*(*x*) (0 < *i* < 100)
	*q_i,t_*	$q_{i,t}=\frac{\sum_{j\in Bi}w_{j,t}}{\left|B_{i}\right|}$ (0 < *i* < 100,0 < *t* < 100)
Parameter of group	*n*	*n* = 100
	*l*	*l* = 2
Parameters of innovation	*μ*_0_	*μ*_0_ = 0.1, 0.3, 0.5, 0.7, 0.9

**Table 2:** The values of *R_t_* based on different σ and $\mu_{0}$.

σ	$\mu_{0}$				
	0.1	0.3	0.5	0.7	0.9
*σ =* 0.00001	0.74	0.74	0.76	0.75	0.73
σ = 0.3	0.72	0.73	0.72	0.76	0.76
σ = 0.5	0.71	0.68	0.77	0.77	0.82
σ = 0.7	0.52	0.61	0.73	0.88	0.97
*σ =* 0.99999	0.33	0.35	0.74	1	1

**Table 3:** The time T based on different σ and $\mu_{0}$.

σ	$\mu_{0}$				
	0.1	0.3	0.5	0.7	0.9
*σ =* 0.00001	3.7	3.66	4.2	3.86	3.44
σ = 0.3	3.6	4.18	3.84	3.9	3.98
σ = 0.5	3.7	3.92	4.04	3.96	3.74
σ = 0.7	2.28	3.44	4.4	4.36	4.36
*σ =* 0.99999	0	0.16	5.06	2.3	1.52
